# Lipid-lowering property of *Clausena anisum-olens* in hypercholesterolemic rats

**DOI:** 10.1080/13880209.2016.1260596

**Published:** 2017-01-31

**Authors:** Anbel M. Bautista, Mario A. Tan, Jovencio M. Apostol

**Affiliations:** a The Graduate School, University of Santo Tomas, Manila, Philippines;; b Research Center for the Natural and Applied Sciences, University of Santo Tomas, Manila, Philippines;; c College of Science, University of Santo Tomas, Manila, Philippines;; d Faculty of Pharmacy, University of Santo Tomas, Manila, Philippines

**Keywords:** Cholesterol, hypercholesterolemia, triglyceride, LDL, HDL, rutaceae

## Abstract

**Context:***Clausena anisum-olens* (Blanco) Merr. (Rutaceae) is a medicinal shrub which has been reported to have various pharmacological uses. No study regarding the effects of *C. anisum-olens* on cholesterol-lowering has been reported.

**Objective:** The effects of the ethanol extract of *C. anisum-olens* leaves on the cholesterol level of hypercholesterolemic rats were evaluated.

**Materials and methods:** Acute oral toxicity of the extract (175, 550 and 2000 mg/kg) was determined using female Sprague-Dawley rats, as described in OECD 425 Main test guidelines. The lipid-lowering assay utilized 30 male Sprague-Dawley rats divided into five groups (A–E). Triton X-100 was administered to induce hypercholesterolemia. After hypercholesterolemia induction, oral treatment of Atorvastatin and crude ethanol extract was given daily to the treatment groups for 14 days. The total cholesterol, triglycerides, HDL and LDL were determined before induction, after induction, after first week of treatment and after second week of treatment.

**Results:** Acute oral toxicity showed the crude extract is nontoxic up to 2000 mg/kg. The lipid-lowering assay indicated reduction of serum cholesterol (87.21 ± 5.10 mg/dL), triglycerides (58.09 ± 4.10 mg/dL) and LDL (27.82 ± 4.11 mg/dL) for 200 mg/kw extract. Reduction in serum cholesterol (74.72 ± 3.64 mg/dL), triglycerides (52.79 ± 2.98 mg/dL) and LDL (12.06 ± 5.51 mg/dL) were observed for 400 mg/kg group. The result is comparable to Atorvastatin, which showed serum cholesterol (80.90 ± 9.72 mg/dL), triglycerides (55.94 ± 7.19 mg/dL) and LDL (22.09 ± 7.60 mg/dL) reduction.

**Discussion and conclusion:** The crude extract of *C. anisum-olens* proved to be useful in lowering of cholesterol.

## Introduction

Coronary artery disease is among the common causes of morbidity and mortality in the Philippines and worldwide. A report released in 2012 revealed that over three million deaths or 26% of total deaths per year have been associated with this condition (WHO 2015). The major risk factor for coronary artery disease is atherosclerosis. This is caused by the presence of high concentration of cholesterol in the serum, particularly the low-density lipoprotein (Deepa & Varalakshmi 2005). Low-density lipoprotein is recognized to undergo oxidative modifications in the artery wall, thus causing coronary artery disease (De Zwart et al. 1999). Statin or HMG-CoA reductase inhibitors have been the drug of choice, such as Atorvastatin, in treating cardiovascular diseases. These drugs possess a favourable safety profile. However, like all other available medications, it also potential in having adverse effects which includes reported cases of muscle adverse effects (Golomb & Evans 2008). Thus, researchers are now geared towards finding substances that can possibly lower cholesterol, and at the same time, can be administered over long periods of time with very minimal adverse effects.

Both the endemic and indigenous Philippine plants are currently under intensive investigation for potential medicinal uses. Among these plants is *Clausena anisum-olens* (Blanco) Merr. (Rutaceae). This shrub is cultivated in the Philippines and other parts of South East Asia. Its aerial parts have been reported to be effective for dysentery and arthritis (Wang et al. 2009). Phytochemical studies on the genus *Clausena* revealed the presence of alkaloids, terpenes, amides, and coumarins (Khan & Naqvi 1988; Yang et al. 1988; Wu et al. 1996; Wang et al. 2008; Maneerat et al. 2010). Pharmacological studies on a variety of *Clausena* species showed anti-trichomonal, anti-diabetic, anti-inflammatory, hepatoprotective, antioxidant, immunomodulatory and anti-cancer properties (Ojewole 2002; Manosroi et al. 2003; Adebajo et al. 2009). Taking into consideration the increasing incidence of coronary artery disease and utilizing the available medicinal plants in the Philippines, this paper reports on the lipid-lowering effects of the crude extract of *C. anisum-olens* utilizing the Triton X-100-induced hypercholesterolemia animal model.

## Materials and methods

### General considerations

Analytical grade ethanol was used in the preparation of the crude extract. Triton X-100 (Sigma Aldrich, Singapore) was used to induce the cholesterol level and the standard drug Atorvastatin Calcium (Pfizer) was used as the positive control for lipid lowering assay. The cholesterol testing kits (Plate 2), cholesterol liquicolour (HUMAN Diagnostics, Wiesbaden, Germany), triglycerides liquicolour mono (HUMAN Diagostics, Germany) and high-density lipoproteins (HUMAN Diagostics, Germany) were acquired from Biocare Health Resources (Quezon City, Philippines).

### The Plant material

Fresh leaves of *C. anisum-olens* were collected from a single shrub in Bataan, Philippines (14.7933° N, 120.4761° E) in March 2015. It was correctly identified and authenticated by Mr. Danilo Tandang of the Botany Division of the National Museum of the Philippines with voucher specimen no. 1331.

Air-dried, ground leaves of *C. anisum-olens* (2 kg) were percolated with 95% ethanol (9 L) for four consecutive days. The combined filtrate was concentrated under reduced pressure to obtain the crude ethanol extract (150 g). The crude extract was kept in an amber coloured bottle, stored in a freezer and was used for the succeeding experiments.

### Experimental animals

The pharmacological study protocol (UST-IACUC Code number RC2014-1421112) was approved by the Institutional Animal Care and Use Committee (IACUC), Research Center for Natural and Applied Sciences, University of Santo Tomas, and was further issued by the Philippine Bureau of Animal Industry and Animal Research Permit. The experiments were performed in accordance with the rules and regulations provided by the institutions mentioned above.

Female Sprague-Dawley rats were used to determine the acute oral toxicity following the methods described in OECD 425 main test guidelines while male Sprague-Dawley rats were used to determine the effects of the crude ethanol extract of *C. anisum-olens* on cholesterol level.

Six female and thirty male adult Sprague-Dawley rats (6–8 weeks old; 150–250 g in weight) were used for the determination of acute oral toxicity (female rats) and assay of the lipid-lowering property of the crude ethanol extract (male rats). The animals were sheltered in the UST Research Center for Natural and Applied Sciences Animal House under environmentally controlled conditions of 22 ± 3 °C and 12 h light/dark cycle. The animals had free access to water and conventional rodent chow pellets (Purina Mills, Manila, Philippines). They were acclimatized at least 1 week before the experiments. After the experiments, the animals were euthanized by the carbon dioxide chamber and subjected to gross necropsy by a licensed veterinarian.

### Acute oral toxicity (OECD 425 Main Test Guidelines)

The acute oral toxicity of the crude ethanol extract of *C. anisum-olens* was determined using the up-and-down dose method described in the OECD 425 Main Test Guidelines. Six female Sprague-Dawley rats were fasted overnight prior to treatment and subsequently administered with fixed doses of the ethanol crude extract (175, 550, and 2000 mg/kg). The doses of the crude ethanol extract of *C. anisum-olens* were prepared by dissolving the extract in 2% Tween 80 vehicle. The first animal was given a 175 mg/kg dose of the extract. When the animal survived after 48 h, the next dose of the extract of 550 mg/kg was given to the second animal. When the second animal survived after 48 h, the next animal was given a dose of 2000 mg/kg (upper bound dose). The testing was terminated until the last three animals survived at the upper bound dose and all of the animals were observed up to 14 days. One female rat served as a control. The animals were sacrificed and a licensed veterinarian conducted gross necropsy and post toxicity test procedures.

## *In vivo* cholesterol lowering assay

Male Sprague-Dawley rats (30) were acclimatized for a period of 7 days prior to experimentation. The rats were grouped into 5 with 6 rats in each group (Groups A–E). Group A served as the normal control group and did not receive the inducing agent. Groups B–E were given Triton X-100 (Rachh et al. 2010) at a dose of 100 mg/kg through intraperitoneal administration to induce hypercholesterolemia. The animals were fasted overnight prior to this procedure. Saline solution was used as the vehicle for the administration of Triton X-100. After successful induction of hypercholesterolemia as indicated by a significant mean difference in serum cholesterol levels of treated groups when compared with the normal group (Table 1), oral treatment of the crude ethanol extract of *C. anisum-olens* and Atorvastatin was given daily up to 14 days.

**Table 1.** Mean serum cholesterol levels (mg/dL) of normal and hypercholesterolemic rats.

Treatment Groups	Baseline X ± SD	Post-induction X ± SD	Week 1 X ± SD	Week 2 X ± SD
Group A (Normal control)	66.53 ± 7.86	89.57 ± 5.44^a^	94.60 ± 3.41^ab^	90.36 ± 9.56^b^
Group B (Negative control – NSS)	71.46 ± 1.72	168.14 ± 19.67^b^	157.05 ± 2.61^c^	141.09 ± 8.02^c^
Group C (Positive control – Atorvastatin)	67.03 ± 5.51	159.05 ± 9.59^b^	102.64 ± 8.81^b^	80.90 ± 9.72^ab^
Group D (200 mg/kg crude extract)	71.26 ± 7.57	183.73 ± 28.05^b^	96.36 ± 1.83^b^	87.21 ± 5.10^ab^
Group E (400 mg/kg crude extract)	66.31 ± 4.19	162.39 ± 23.31^b^	87.31 ± 3.67^a^	74.72 ± 3.64^a^

Data are presented as mean ± SD, *n* = 6 per group. The values in the same column with different superscripts letters are statistically significantly (*p* < 0.05).

During the bioassay, Group A received no treatment. Group B received only normal saline solution and was considered the negative control group. Group C received the standard drug Atorvastatin calcium (Pfizer) at an oral daily dose of 10 mg/kg through oral gavage and was considered the positive control group. Groups D and E received the crude ethanol extract through oral gavage at doses of 200 and 400 mg/kg, respectively. All groups had free access to water and food during the bioassay.

Blood samples were extracted four times through tail clipping method: prior to induction of hypercholesterolemia (baseline); after the induction of hypercholesterolemia (post-induction); 7th day of oral treatment (Week 1) and 14th day of the oral treatment (Week 2). The blood samples were centrifuged to obtain the serum. The sera were then subjected to biochemical assay using enzymatic colorimetric methods and read at 550 nm with the Corona Microplate Reader SH-1000 (Hitachi).

Three representatives in each group were sacrificed and subjected to gross necropsy and post toxicity tests by a licensed veterinarian. The remaining rats were euthanized by carbon dioxide chamber and disposed properly in accordance with the approved protocol of the IACUC.

The cholesterol parameters were computed using the formula given below:

Total cholesterol

TC= 200 × (Absorbance of the sample/Absorbance of the standard)

Triglyceride

TG =200 × (Absorbance of the sample/Absorbance of the standard)

HDL cholesterol (semi micro method)

HDL-c = 175 × (Absorbance of the sample/Absorbance of the standard)

### LDL cholesterol using Friedewald equation

LDL-c = TC – HDL − (TG/5)

### Statistical treatment

The data gathered were recorded as mean ± SEM. The statistical significance of differences between groups was determined by two-way analysis of variance (two-way ANOVA) using SPSS 17.0 software (Chicago, IL). Mean values were considered statistically significant when *p* < 0.05 using Tukey’s HSD *post hoc* test.

## Results and discussion

### Acute oral toxicity

Assessment of the acute oral toxicity of the crude ethanol extract of *C. anisum-olens* utilizing the OECD 425 Main test revealed that it is safe and nontoxic to use the dose of 175–2000 mg/kg. There were also no notable variations in behavioural pattern of the female Sprague-Dawley rats during the 14-day observation period. Also, prominent signs of toxicity were not observed after the administration of the extract. Moreover, the post toxicity and gross necropsy study showed that all vital organs such as the liver, kidneys, heart and gastrointestinal tract were within normal limits as indicated in the histopathological results analyzed by a licensed veterinarian.

### *In vivo* cholesterol-lowering assay

Male Sprague-Dawley rats were used in the *in vivo* cholesterol-lowering assay was done to avoid the effects of hormonal factors in developing metabolic diseases, i.e., type 2 diabetes and insulin resistance (Bruns & Kemnitz, 2004). The 10 mg/kg dose of the Atorvastatin was the chosen as adopted from established protocol for the determination of lipid-lowering property of plant samples in rats (Gundaramanju et al., 2014). The 200 mg/kg dose served as the lower limit, while the 400 mg/kg was the upper limit dose. The serum cholesterol, triglycerides, high-density lipoproteins and low-density lipoproteins were monitored weekly for two weeks to determine if the effect of the crude extract is time-dependent.

Table 1 shows the mean serum cholesterol level of normal and hypercholesterolemic rats over the bioassay period. The baseline data revealed that there was no significant difference among all experimental groups before induction of hypercholesterolemia. Three (3) days after the induction of hyperlipidemia for Groups B–E (using 100 mg/kg of Triton X-100 administered once through intra-peritoneal injection), the mean values were significantly different compared to the normal control Group A indicating a successful induction of hyperlipidemia for Groups B–E.

After Weeks 1 and 2 of treatment, data showed that there was a significant difference among the mean values of the groups. Total cholesterol level for treatment groups C (80.90 ± 9.72 mg/dL), D (87.21 ± 5.10 mg/dL) and E (74.72 ± 3.64 mg/dL) were significantly different from Group B (141.09 ± 8.02 mg/dL). Table 1 also shows the baseline total cholesterol level of Groups C–E are statistically non-significant to Group A. Therefore, the crude ethanol extract of *C. anisum-olens* at daily doses of 200 and 400 mg/kg decreased the total cholesterol values in hypercholesterolemic rats after one and two weeks of treatment. This also demonstrated that the crude ethanol extract exhibited cholesterol-lowering property similar to the expected effect of atorvastatin.

Table 2 shows the effect of atorvastatin and crude extract of *C. anisum-olens* on serum HDL of hypercholesterolemic rats. Baseline level showed that there was no significant difference among the treatment groups. Three (3) days after induction with Triton X-100, the mean values were significantly different. Thus, the values would indicate that induction of hypercholesterolemia was successful as depicted by a decrease in HDL levels among rats in Groups B–E.

**Table 2.** Mean serum HDL levels (mg/dL) of normal and hypercholesterolemic rats.

Treatment groups	Baseline X ± SD	Post-induction X ± SD	Week 1 X ± SD	Week 2 X ± SD
Group A (Normal control)	47.29 ± 2.01	47.96 ± 5.03^c^	39.44 ± 5.52^a^	41.46 ± 3.33^a^
Group B (Negative control – NSS)	51.45 ± 1.70	43.01 ± 5.42^bc^	39.50 ± 4.15^a^	40.19 ± 3.20^ab^
Group C (Positive control – Atorvastatin)	47.92 ± 3.05	38.53 ± 2.58^ab^	45.42 ± 3.00^b^	47.62 ± 2.08^bc^
Group D (200 mg/kg crude extract)	47.89 ± 2.52	36.91 ± 4.63^ab^	47.57 ± 5.17^b^	47.77 ± 1.91^bc^
Group E (400 mg/kg crude extract)	46.76 ± 4.35	32.97 ± 2.34^a^	45.80 ± 5.20^b^	52.09 ± 5.53^c^

Data are presented as mean ± SD, *n* = 6 per group. The values in the same column with different superscripts letters are statistically significantly (*p* < 0.05).

After Week 2 of treatment, data showed that groups given with the crude ethanol extract at doses 200 and 400 mg/kg and atorvastatin (10 mg/kg) were able to regain the HDL levels similar to the baseline levels. The crude ethanol extract increased the HDL values in hypercholesterolemic rats after two weeks of treatment.

Table 3 shows the effect of the crude ethanol extract of *C. anisum-olens* on serum triglycerides as compared to Atorvastatin-treated group and the negative control group. The baseline level showed that there was no significant difference among the groups before induction. Three days after the induction of hypercholesterolemia, the mean values were significantly different, thus indicating that the desired outcome was achieved.

**Table 3.** Mean serum triglyceride levels (mg/dL) of normal and hypercholesterolemic rats.

Treatment groups	Baseline X ± SD	Post-induction X ± SD	Week 1 X ± SD	Week 2 X ± SD
Group A (Normal control)	84.61 ± 7.75	88.00 ± 5.53^a^	65.55 ± 4.85^a^	56.67 ± 5.29^a^
Group B (Negative control – NSS)	88.53 ± 6.58	147.41 ± 20.89^b^	92.96 ± 13.49^b^	89.63 ± 6.84^b^
Group C (Positive control – Atorvastatin)	84.12 ± 15.62	155.31 ± 41.41^b^	66.94 ± 14.84^a^	55.94 ± 7.19^a^
Group D (200 mg/kg crude extract)	75.13 ± 6.22	136.17 ± 37.04^ab^	61.02 ± 9.75^a^	58.09 ± 4.10^a^
Group E (400 mg/kg crude extract)	89.29 ± 5.19	125.81 ± 37.45^ab^	55.50 ± 5.89^a^	52.79 ± 2.98^a^

Data are presented as mean ± SD, *n* = 6 per group. The values in the same column with different superscripts letters are statistically significantly (*p* < 0.05).

After Weeks 1 and 2 of treatment, data showed that there was a significant difference among the mean values of the negative control compared with those of the treatment groups. Triglyceride levels of atorvastatin-treated group C (55.94 ± 7.19 mg/dL) were comparable with Groups D (58.09 ± 4.10 mg/dL) and E (52.79 ± 2.98 mg/dL). Therefore, the crude extract of *C. anisum-olens* decreased the triglyceride values of hypercholesterolemic rats after two weeks treatment with comparable effects to atorvastatin. After two weeks of treatment, the crude extract seemed to normalize the triglyceride levels similar to the baseline values. Elevated serum triglyceride levels accelerated atherogenesis and, therefore, the significantly lowered serum triglyceride levels will be advantageous if preventive action against heart attack is a goal of therapy (de Guzman et al. 2013).

Table 4 shows the serum LDL of normal and hypercholesterolemic rats. Baseline level showed that there was no significant difference among the treatment groups prior to induction of hypercholesterolemia. After induction of hypercholesterolemia and treatment with atorvastatin (Group C) and the crude ethanol extract (Group D and E), data revealed significant difference on the mean values between Group B (82.97 ± 8.73 mg/dL) and Groups C, D and E. No significant difference was noted between Groups C (22.09 ± 7.60 mg/dL) and D (27.82 ± 4.11 mg/dL). Atorvastatin at 10 mg/kg dose and the crude extract of *C. anisum-olens*, both 200 and 400 mg/kg, were able to reduce LDL after 2 weeks of treatment. The main determinant in the development of atherosclerosis is high levels of low-density lipoproteins. The oxidation of LDL induces inflammatory responses by producing leukocytes and cytokine in endothelial tissues. Oxidation of LDL produces reactive oxygen species that are noxious. Therefore, a plant extract that can diminish both serum LDL and triglyceride levels is beneficial in regressing atherosclerosis.

**Table 4.** Mean serum LDL levels (mg/dL) of normal and hypercholesterolemic rats.

Treatment groups	Baseline X ± SD	Post-induction X ± SD	Week 1 X ± SD	Week 2 X ± SD
Group A (Normal control)	2.31 ± 8.46	24.01 ± 6.48^a^	42.05 ± 7.22^b^	37.57 ± 6.42^b^
Group B (Negative control – NSS)	2.30 ± 3.83	95.65 ± 24.94^b^	98.96 ± 3.87^c^	82.97 ± 8.73^c^
Group C (Positive control – Atorvastatin)	2.28 ± 3.74	89.46 ± 17.40^b^	43.83 ± 9.28^b^	22.09 ± 7.60^a^^b^
Group D (200 mg/kg crude extract)	8.35 ± 9.67	119.59 ± 18.28^b^	36.59 ± 3.47^a^^b^	27.82 ± 4.11^b^
Group E (400 mg/kg crude extract)	1.69 ± 7.97	104.27 ± 20.97^b^	30.41 ± 5.85^a^	12.06 ± 5.51^a^

Data are presented as mean ± SD, *n* = 6 per group. The values in the same column with different superscripts letters are statistically significantly (*p* < 0.05).

It is always beneficial to use a drug that decreases serum total cholesterol, triglycerides and low-density lipoprotein levels and elevates serum HDL levels (de Guzman et al. 2013). However, this study showed that cholesterol levels only serve as a surrogate indicator for the development of atheroschlerosis and other related cardiovascular conditions. Therefore, a comprehensive investigation about the direct effect of the crude extract on the formation of atheroschlerosis must be focussed in future studies.

## Conclusion

This study is the first report on the effect of the crude ethanol extract of *C. anisum-olens* to reduce the serum cholesterol, triglycerides and low-density lipoproteins in hypercholestoremic male Sprague-Dawley rats. Considering the variation of associated pharmacological properties, *C. anisum-olens* has a strong potential in its application in the nutraceutical industry.
